# Resistance between channels may lead to increased action potential efficiency

**DOI:** 10.1186/1471-2202-13-S1-P77

**Published:** 2012-07-16

**Authors:** Jack H Wilson, Sorinel A Oprisan

**Affiliations:** 1 Department of Physics, College of Charleston, Charleston, SC 29466, USA

## 

The energy efficiency of an action potential (AP) is typically defined as the amount of capacitive current used to depolarize the membrane relative to the amount of ATP necessary to reset the displacement of sodium and potassium ions that occurs during the AP. Research in recent years suggests that at least some neurons have been optimized for energy-efficiency, which falls within the framework that information storage and processing in neural systems occurs in a manner that minimizes energy consumption [2]. Improvements in AP energy efficiency typically occur by decreasing the overlap in sodium and potassium currents. Previous studies have shown that this can be done via manipulation of the gating kinetics and conductance densities [1,3,4] of fast, voltage-gated sodium (Na_V_) channels, and slower repolarizing potassium (K_DR_) channels, and that the optimal manipulation of these factors is model-dependent [4]. Biophysical models have been a common means of making these determinations.

Biophysical models, such as those used to predict energy efficiency, typically assume that the only significant differences in membrane voltage are along the long axis of the neuron: non-membrane current orthogonal to the long axis is assumed to be zero. Implicit in this assumption is that there is zero resistance between any two individual channels in that section. This study considers the impact of including a non-zero “interchannel” resistance between Na_V_ and K_DR_ channels that may otherwise be included in the same compartment. This is done by expanding single compartment Hodgkin-Huxley models into two compartments representing membrane regions immediately surrounding Na_V_ and K_DR_ channels, with a degree of resistance between these two compartments. Simulations were run for a range of interchannel resistances and relative areas of the two compartments, and the energy efficiency was measured using multiple previously established measures.

Results of these simulations suggest that the energy-efficiency of an action potential may be optimized by varying the relative area surrounding Na_V_ and K_DR_ channels as a function of the degree of resistance between these two channel types. This model predicts that the energy efficiency of action potentials may be smaller than predicted by most biophysical models and suggests that the relative concentrations and single-channel conductances of depolarizing Na and repolarizing K channels may be subject to selection based upon energy consumption. The model also displays an upper bound for the degree of resistance between sodium and potassium channels, beyond which the neuron fails to completely repolarize. Results from this simulation have also shown some disagreement in commonly-used measures of AP energy efficiency.

## References

[B1] Alle H Roth A Geiger J Energy-efficient action potentials in hippocampal mossy fibers Science 2009 325 doi:10.1126/science.1174331 10.1126/science.1174331 19745156

[B2] Hasentaub A Otte S Callaway E Sejnowski TJ Metabolic cost as a unifying principle governing neuronal biophysics Proc Nat Acad Sci USA 2010 107 27 12329 12334 10.1073/pnas.0914886107 20616090 PMC2901447

[B3] Schmidt-Heiber C Bischofberger J Fast sodium channel gating supports localized and efficient axonal action potential initiation J Neurosci 2010 30 30 10233 10242 10.1523/JNEUROSCI.6335-09.2010 20668206 PMC6633381

[B4] Sengupta B Stemmler M Laughlin S Niven J Action potential energy efficiency varies among neuron types in vertebrates and invertebrates PLoS Comp Bio 2010 6 7 e1000,840 doi:10.1371/journal.pcbi.1000840 10.1371/journal.pcbi.1000840 PMC2895638 20617202

